# Supplementary material for: Complexome profiling of the Chlamydomonas psb28 mutant reveals TEF5 as an early PSII assembly factor
Source: Plant Cell. 2025 Mar 17;37(6):koaf055. doi: 10.1093/plcell/koaf055 (PMC12164590; doi:10.1093/plcell/koaf055)
Supplement: koaf055_Supplementary_Data [file koaf055_supplementary_data.zip › psb28_interactive.zip!/plots/intensity/qualityPlots/peptides/NUO21_Cre06.g267200.t1.2.html]

### Cre06.g267200.t1.2

Trivial name: NUO21  
SeqCoverage [median]: 59.50  
Mass: 13.45 kDa  
  
 

| Sequence | MissedCleavage | Mass [Da] | wtIntensity | muIntensity | Score |
| --- | --- | --- | --- | --- | --- |
| EVAANLPHVMQR | 0 | 1363.70 | 24054.67 | 17232.49 | 78.65 |
| LMGFKENSK | 1 | 1052.53 | 0.00 | 1174.08 | 44.32 |
| SGLLWGMSDSAHR | 0 | 1415.66 | 4246.77 | 2746.77 | 70.44 |
| TWPEPPNYYEAPK | 0 | 1590.74 | 820.61 | 2086.12 | 51.93 |
| VTLENPTFPIINPDPNITDAVTNMR | 0 | 2781.40 | 7136.25 | 0.00 | 103.71 |
|  |  |  | 36258.3 | 23239.5 |  |
